# Epigenetics of Subcellular Structure Functioning in the Origin of Risk or Resilience to Comorbidity of Neuropsychiatric and Cardiometabolic Disorders

**DOI:** 10.3390/ijms19051456

**Published:** 2018-05-14

**Authors:** Carlos Manuel Zapata-Martín del Campo, Martín Martínez-Rosas, Verónica Guarner-Lans

**Affiliations:** Department of Psychiatry, Instituto Nacional de Cardiología “Ignacio Chávez”, Ciudad de México 14080, Mexico; carlos.zapata@cardiologia.org.mx (C.M.Z.-M.d.C.); matin.martinez@cardiologia.org.mx (M.M.-R.)

**Keywords:** epigenetics, mitochondria, endoplasmic reticulum stress, telomere length, DNA repair, neuropsychiatric disorders, cardiometabolic disorders

## Abstract

Mechanisms controlling mitochondrial function, protein folding in the endoplasmic reticulum (ER) and nuclear processes such as telomere length and DNA repair may be subject to epigenetic cues that relate the genomic expression and environmental exposures in early stages of life. They may also be involved in the comorbid appearance of cardiometabolic (CMD) and neuropsychiatric disorders (NPD) during adulthood. Mitochondrial function and protein folding in the endoplasmic reticulum are associated with oxidative stress and elevated intracellular calcium levels and may also underlie the vulnerability for comorbid CMD and NPD. Mitochondria provide key metabolites such as nicotinamide adenine dinucleotide (NAD+), ATP, α-ketoglutarate and acetyl coenzyme A that are required for many transcriptional and epigenetic processes. They are also a source of free radicals. On the other hand, epigenetic markers in nuclear DNA determine mitochondrial biogenesis. The ER is the subcellular organelle in which secretory proteins are folded. Many environmental factors stop the ability of cells to properly fold proteins and modify post-translationally secretory and transmembrane proteins leading to endoplasmic reticulum stress and oxidative stress. ER functioning may be epigenetically determined. Chronic ER stress is emerging as a key contributor to a growing list of human diseases, including CMD and NPD. Telomere loss causes chromosomal fusion, activation of the control of DNA damage-responses, unstable genome and altered stem cell function, which may underlie the comorbidity of CMD and NPD. The length of telomeres is related to oxidative stress and may be epigenetically programmed. Pathways involved in DNA repair may be epigenetically programmed and may contribute to diseases. In this paper, we describe subcellular mechanisms that are determined by epigenetic markers and their possible relation to the development of increased susceptibility to develop CMD and NPD.

## 1. Introduction

Epigenetic modifications to DNA such as methylation and/or histone modifications including phosphorylation, lysine and arginine methylation or acetylation are potentially important mechanisms underlying the relations between the genome and environmental exposures. They might be responsible for the comorbid susceptibility to cardiometabolic and neuropsychiatric disorders (CMD and NPD) [1,2]. The term comorbidity refers to the presence of one or more coexistent diseases in addition to a primary disorder. CMD are among the most important causes of premature death and chronic disability in the world [3], and NPD (including mental and psychotropic substance abuse diseases) are the main causes of years lived with disability worldwide [4]. The association between CMD and NPD diseases is bidirectional; neuropsychiatric symptoms are commonly present in patients with chronic systemic diseases [5], and different types of psychiatric disorders including stress-related disorders, depression, emotional incontinence, delusions and hallucinations are frequently observed after the occurrence of CMD, particularly coronary artery disease [5,6]. NPD (mainly stress-related disorders) are independent risk factors to develop CMD including coronary artery disease, often triggering the disease [7]. Subcellular structures may play a role in the vulnerability to this comorbidity acting as pathological centers for the appearance of common disorders and/or in their comorbid and probably genetic or epigenetic genesis. In this paper, we support the epigenetic contribution of the mitochondria, endoplasmic reticulum (ER) and nucleus in the development of the comorbid process of CMD and NPD.

Biochemical signals contribute to the establishment of specific transcriptional programs, thus regulating the appearance of epigenetic cues. The ways in which cell signaling pathways can interact with epigenetic elements appear to be varied and complex. Reactive oxygen species (ROS) and Ca^2+^ might mediate the integration of subcellular organelles and epigenetic cues. There are also ATP-dependent chromatin remodeling complexes that might be altered when mitochondria are not working correctly. These remodeling complexes modulate different chromatin configurations and gene expression. Several biochemical signaling pathways that include mitogen-activated protein kinase (MAPK), the wingless-related integration site pathway (Wnt), Notch, Janus kinases (JAKs)-signal transducer and activator of transcription proteins (STATs), the c-Jun N-terminal kinase (JNK) pathway, nuclear factor kappa-light chain enhancer of activated B cells (NF-κB) and protein kinase A (PKA) pathways have also been found to participate in the integration of cell signaling and epigenetics [8]. In addition, complexes that were first described in the fruit fly such as the protein polycomb group (PcG) and the trithorax group (TrxG) have also been associated with different covalent histone modifications. The PcG and TrxG proteins represent two of the most important epigenetic regulators. Integration of cell signaling and epigenetics is beginning to be considered as the next step for the comprehension of many complex processes [8].

Modifications of the genomic expression of proteins controlling mitochondrial function, protein folding in the ER and nuclear processes such as telomere length determination and DNA repair might be, in part, the basis of the common susceptibility (or resilience) to develop CMD and NPD. Particularly, adverse early life experiences significantly contribute to the possibility of developing CMD and NPD later in life in when adulthood is reached [9]. Neurodegenerative diseases have many known environmental risk factors, and there is evidence that early life exposure can increase the risk of the condition later in life. In a similar manner, there are known effects of maternal nutrition on metabolic syndrome and cardiovascular risk in the offspring when they reach adulthood [10]. Neurodegenerative diseases might influence more than one generation since inhibitors of histone-modifying enzymes can cause DNA methylation changes that are inherited and associated with disease phenotypes [2,10]. This is an area of research that is likely to grow and yield interesting insights in the near future [11]. Furthermore, epigenetics is a new area for the development of new alternatives that may return the chromatin to the initial state before being remodeled by environmental factors [12,13,14,15,16]. Challenges for modifying epigenetic cues are related to the comorbidity of CMD and NPD including lifestyle changes, non-pharmacological and pharmacological treatments [9]. DNA modifications might function as novel biological biomarkers of exposure, risk or progression of disease. In this paper, we describe subcellular mechanisms that are determined by epigenetic markers and their possible relation to the development of increased susceptibility or resilience to develop CMD and NPD. The information discussed is summarized in Table 1.

## 2. Mitochondria

Mitochondria constitute central players in cellular energetic metabolism since they generate most of the cellular ATP. Besides, they participate in the response to ROS, sensing of nutrients and in crosstalk with the nucleus [47,48,49,50,51]. Mitochondria also play an important role in functions such as apoptosis, control of cytosolic Ca^2+^ levels, lipid homeostasis, steroid synthesis, generation of Fe-S (iron-sulfur) centers, heme synthesis, innate immune response and metabolic cell signaling [47,48,49,50,51].

Through the generation of metabolites by the tricarboxylic acid cycle, mitochondria participate in epigenetic alterations, mitochondria-nuclear signaling, biogenesis, fission/fusion and mitophagy. Mitochondria and nucleus inter-communicate, and this crosstalk is central to integrate mitochondrial functions with other cellular structures, in proper nuclear functions and in determining the way in which the environmental factors impact the cell [19].

### 2.1. Mitochondria/ Nuclear DNA Interactions

The evolution of the eukaryotic cell was dramatically changed by the endosymbiosis of an energy-limited ancient cell with an oxidative α-proteobacterium about two billion years ago. Energy delivery for the metabolic process was significantly increased by this process, and the nucleus-mitochondria interactions began [47,48,49,50,51]. Energy production by mitochondria contributed to providing the ATP required for improved cell functions. For the next 1.2 billion years, the nucleus-cytosol specialized in structure, while energy production was mostly the business of mitochondria. High energy was provided by phosphorylation by ATP, acetylation by acetyl coenzyme A (acetyl-CoA), deacetylation by nicotinamide adenine dinucleotide (NAD+) and methylation by S-adenosyl-methionine [47,48,49,50,51] and a discrete amount delivered by glycolysis, except for the erythrocyte, for which glycolysis is the unique source. This subcellular specialization rendered the possibility of multicellularity [17,18], species radiation [18] and environmental adaptation.

At the same time, the nucleus acquired mechanisms for regulating mitochondrial growth and replication [17,18]. Additional mechanisms had to evolve to allow for the coordinated expression of the mitochondrial genes, which were exchanged with nuclear DNA, according to the nuclear requirements of energy for growth and reproduction [17,18]. Thus, inter-chromosomal coordinated transcriptional regulation was the result.

### 2.2. Cross-Talk between Mitochondria and Nucleus and Epigenetic Marks in Nuclear DNA

**Figure 1:**
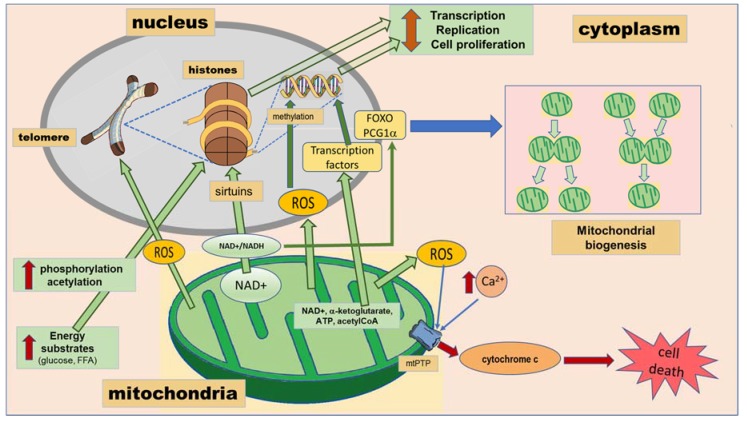
Crosstalk between the mitochondria and the nucleus resulting in epigenetic markers. Green arrows correspond to signals emitted by mitochondria to influence the nucleus and therefore altering the telomeres, the nucleosome and the DNA. Reactive oxygen species (ROS) mediate damage. Free fatty acids (FFA) and glucose lead to phosphorylation and acetylation of histones. Nicotinamide adenine dinucleotide (NAD+) regulates sirtuins that are histone deacetylases. The blue arrow corresponds to signaling from the nucleus to mitochondrial biogenesis. Peroxisome proliferator-activated receptor gamma coactivator 1 alpha (PCG1α) and forkhead transcription factors (FOXO) regulate mitochondrial biogenesis. The red arrows correspond to the pathway leading to cell death. Mitochondrial permeability transition pore (mtPTP) activation leads to apoptosis.

There is increasing evidence that suggests a mitochondrial role in modulating the epigenome. Mitochondria provide key metabolites such as NAD+, ATP, α-ketoglutarate and acetyl-CoA that are necessary for numerous transcriptional and epigenetic processes [52,53,54]. Moreover, epigenetic markers in nuclear DNA also determine mitochondrial biogenesis (Figure 1).

### 2.3. Mitochondria Determine Epigenetic Modification Reactions

Phosphorylation-dephosphorylation reactions were the first post-translational modifications that appeared in the regulation of DNA-protein interactions. The main mechanism for generating ATP was substrate phosphorylation in non-photosynthetic organisms before the availability of free oxygen in the biosphere. After the generation of free oxygen by photosynthesis, oxidative phosphorylation became the main system for generating ATP [17,18,55,56]. Thus, mitochondria became the most effective providers of the ATP needed for protein modifications. ATP concentrations increase when substrates are abundant, leading to increased phosphorylation of histones (Figure 1). The proteins would then be repelled from the sugar-phosphate backbone of the DNA, which is negatively charged by the phosphate group; rendering the chromatin less dense for DNA transcription and replication. Phosphorylation is also used to regulate energy availability in relation to enzyme activities, signal transduction pathways and the transcriptional apparatus [17,18].

When carbohydrates and/or fats are plentiful, protein acetylation increases. The positive charge of histones is neutralized by acetylation of lysines, diminishing protein affinity for DNA. Transcription, replication and cell proliferation are increased when histones are acetylated (Figure 1). In contrast, in conditions such as fasting-starvation where carbohydrates and fats are limited, acetyl-CoA levels are reduced; acetylation diminishes; the chromatin condenses; and cellular gene expression, replication and proliferation are stopped. In mammalian cells, citrate derived from glucose through the mitochondrial tricarboxylic acid cycle provides the carbon for acetyl-CoA destined for histone acetylation [17,18].

Reduced energy supplies in conditions such as fasting, in addition to reducing acetyl-CoA levels, also diminish the availability of reducing equivalents, resulting in the oxidation of reduced nicotinamide adenine dinucleotide (NADH) to NAD+. Since starvation produces NAD+, it activates histone deacetylases known as sirtuins to deacetylate the DNA-binding proteins (Figure 1). The change from glycolysis to oxidative phosphorylation may also be mediated by the cytosolic NAD+/NADH levels through sirtuin 1 (Sirt1) (Figure 1). This elevates the positive charge of the chromatin proteins, having as a consequence, chromatin condensation, and inhibition of transcription, replication, growth and proliferation [17,18].

Phosphorylation or acetylation are less versatile for chromatin regulation than DNA methylation. Gene expression and replication in response to energy availability and nutrient supply are also regulated through another macromolecular modification system constituted by S-adenosyl-l-methionine methylation. DNA methylation increases macromolecular interactions by van der Waals forces, diminishing transcription. DNA methylation is also influenced by changes in redox states [57], which may also alter recognition by methyl-binding proteins and therefore alter epigenetic regulation [57]. Mitochondria act as redox sensors modifying energy states in response to the chemical environment and levels of endogenous metabolites, including iron, succinate and ascorbate.

### 2.4. Mitochondrial Biogenesis Is Determined by the Epigenetic Program

Epigenetic programming by environmental factors regulates the biogenesis of mitochondria, which undergo repeated cycles of fission and fusion [58]. These processes mix the mitochondrial inner and outer membranes, combine mitochondria matrices and reallocate the mitochondrial DNAs. The mammalian mitochondrial fusion machinery involves three major proteins, mitofusins 1 and 2 and the optic atrophy-1 protein [59,60,61], while the mitochondrial fission machinery involves dynamin-related protein 1, mitochondrial fission factor and fission protein 1 [62,63,64,65]. 

The increase in mitochondrial biogenesis is promoted by acetylation of forkhead transcription factors (FOXOs) and peroxisome proliferator-activated receptor gamma coactivator 1 alpha (PCG-1α) [18] (Figure 1). PCG1α constitutes the main regulator of mitochondrial biogenesis, and as a result of its activation, targets such as nuclear erythroid-related factors 1 and 2 (Nrf1, Nrf2) and mitochondrial transcription factor A (Tfam) are induced [23]. Sirt1 deacetylates FOXOs and PCG1α when glucose is abundant and cytosolic NAD+ is reduced, rendering these molecules inactive and inhibiting mitochondrial biogenesis (Figure 1). Glucose catabolism by glycolysis reduces cytosolic NAD+ to NADH, while fatty acid oxidation leaves cytosolic NAD+ oxidized, promoting biogenesis [18].

### 2.5. Mitochondrial Biogenesis in Neuropsychiatric and Cardiometabolic Disorders

Many common disorders, including neurodegenerative diseases, type-2 diabetes and cardiovascular diseases, have been associated with altered organellar regulation and mitochondrial dysfunction [19,61,66]. The association of numerous chronic diseases and mitochondrial dysfunction may reflect exogenous insults and deleterious effect of the environmental on mitochondrial vulnerability [19]. Altered mitochondrial biogenesis and mitochondrial fusion and fission processes have been associated with diseases [58]. In addition, each mammalian cell contains hundreds of mitochondria and thousands of mitochondrial DNAs. When mutations surge in mitochondrial DNA, they lead to a state known as heteroplasmy in which there is a mixed population of normal and mutant DNAs. When heteroplasmic cells divide, the two types of DNAs are distributed at random into the daughter cells, generating a genetic drift toward either pure mutant or wild type. In the long term, segregation of the mutant mitochondrial DNAs results in pure mutant or normal populations, termed homoplasmic cells [67,68]. As the percentage of mutant mitochondrial DNAs increases, mitochondrial energetic function decreases. When energy output is not enough for normal tissue function, symptoms of diseases appear, and apoptosis, necroptosis, pyroptosis, or necrosis determine the course of the disease [17,68].

Mitochondria possess a self-destruction system, the mitochondrial permeability transition pore (mtPTP) (Figure 1). As the biochemical health of the mitochondria declines, energy production is reduced, there is increased production of ROS, and Ca^2+^ is released into the cytosol. Calcium taken up by the mitochondrion results in cytochrome c being released and mtPTP activated. When mtPTP is activated, it opens a channel in the mitochondrial inner membrane, the negative electrical potential (Δ*P*) is lost and the cell initiates programmed cell death (apoptosis) (Figure 1) [17].

Mitochondrial dysfunction and oxidative stress have been associated with major psychiatric disorders, including bipolar disorder, schizophrenia, autism, attention deficit-hyperactivity disorder and Alzheimer’s dementia. Mitochondrial dysfunction might induce the vulnerability of brain cells to other disease-specific factors. The impairment in their function may be region specific, and cell susceptibility might vary among different brain cells. Although mitochondrial dysfunction could also be a consequence of habits often found in psychiatric patients such as smoking, drug abuse and disturbed eating and sleeping [69], mitochondrial dysfunctions may be involved in the pathophysiology of many diseases. Moreover, mitochondria could contribute to the process of neural apoptosis [25,70]. Redox-proteomics and epigenomics could also explain the important role of oxidative damage in most psychiatric disorders and could drive towards the identification of targets for the development of new drugs [71].

The expression of mitochondrial genes and nuclear genes that alter mitochondrial number and function are determined by glucocorticoids in neurons [52,53] and thus are linked to anxiety disorders. Glucocorticoid receptors linked to mitochondrial membranes are commonly associated with regulation of the membrane potential [72,73]. Early life stress leads to oxidative stress, increasing glutamate transmission and resulting in mitochondrial damage [74,75].

The pathogenesis of cardiovascular diseases, including myocardial ischemia, cardiomyopathy and heart failure, might also be due to malfunction of the mitochondria [76]. Mitochondria, by regulating metabolic and energy homeostasis, show important changes in type 2 diabetes patients and in obese subjects. Since epigenetic regulation plays a pivotal role in mitochondrial biogenesis, function and dynamics, it has been proposed to participate in the development of these conditions. Exercise, a non-pharmacological treatment for diabetes and obesity, changes DNA methylation of the promoter of PGC1α to facilitate the gene expression that is responsible for mitochondrial biogenesis and function [23]. In conditions where nutrient or energy depletion is present, including fasting, exercise or calorie restriction, the level of cAMP and the ratio of AMP/ATP increase, initiating the signaling cascades of cAMP-dependent protein kinase (PKA)/cAMP response element-binding protein (CREB), AMP-activated protein kinase (AMPK) and Sirt1, which can activate PGC1α [23]. 

Mitochondrial DNA damage, increased production of ROS and respiratory chain dysfunction play critical roles in atherogenesis by affecting endothelial function, vascular smooth muscle cell proliferation or apoptosis [77,78]. Hypercholesterolemia, hyperglycemia, hypertriglyceridemia and aging, which constitute atherosclerosis risk factors, induce mitochondrial dysfunction [77]. Mitochondria are the main intracellular source of ROS, and these species significantly contribute to the initiation and development of atherosclerotic lesions. Mitochondria are also the main targets, when there is an excess ROS triggering inflammatory responses and cell death. Mitochondrial biogenesis and the mitochondrial antioxidant system counteract the effects of ROS. Moreover, mitochondrial abundance is regulated in response to diet and ROS production [77,78]. Mitochondrial damage and lipoperoxidation may be the main mechanisms by which hypercholesterolemia causes the formation of the atherosclerotic lesion [78].

Plaque rupture, which may result in myocardial infarction, stroke and ischemic/reperfusion damage, might also be favored by mitochondrial dysfunction that results in apoptosis. Impaired mitochondrial integrity predisposes to vascular cell growth [79].

Summarizing, mitochondrial function is associated with oxidative stress and excess calcium levels and may also underlie the comorbidity of CMD and NPD. Mitochondria provide key metabolites such as NAD+, ATP, α-ketoglutarate and acetyl-CoA that are co-substrates required for numerous transcriptional and epigenetic processes (Figure 1). Increased mitochondrial calcium concentration causes cytochrome c release and altered membrane potential that eventually trigger cellular death programs (Figure 1). They are an important source of free radicals. Moreover, epigenetic markers in nuclear DNA determine mitochondrial biogenesis. In addition to crosstalk with the nucleus, mitochondria influence the functioning of other organelles, as will be discussed in the next sections. ROS generated through mitochondrial dysfunction accelerate ER malfunction and may promote telomere shortening.

## 3. Endoplasmic Reticulum

The state in which the proteome is kept in functional balance in an organism is protein homoeostasis or proteostasis. Maintaining proteostasis requires the correct functioning of processes such as protein synthesis, degradation and folding. The ER is the subcellular organelle in which proteins are synthesized and folded. Many environmental factors stop the ability of cells to properly fold proteins and modify post-translationally secretory and transmembrane proteins. When this happens, there is an accumulation of misfolded or unfolded proteins in this organelle, leading to ER stress. Cells undergoing ER-stress must restore the protein-folding capacity to survive [80].

**Figure 2:**
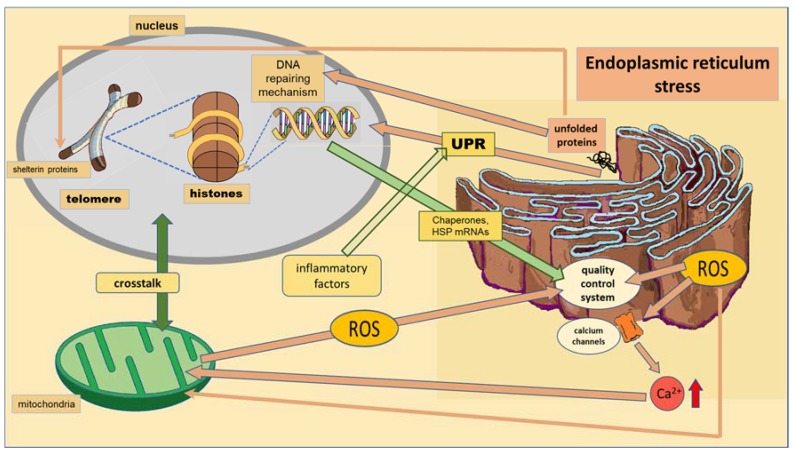
Crosstalk between the endoplasmic reticulum (ER) and the nucleus and mitochondria. The brownish arrows indicate signals coming from ER and the green arrow the signal from the nucleus to ER. ROS mediate the loss of calcium homeostasis that influences the crosstalk between the mitochondria and nucleus. Inflammatory factors enhance the unfolded protein response (UPR) generated by ER stress.

When unfolded or misfolded proteins accumulate in the ER, an intracellular signaling pathway called the unfolded protein response (UPR) induces processes that try to restore ER homeostasis. The UPR transmits information to the cytosol and nucleus, leading to the upregulation of genes encoding ER chaperones, heat shock proteins (HSP) and initiation of the ER quality control system (Figure 2). Ubiquitously present small HSP show anti-apoptotic and anti-inflammatory activities, protecting cells from stress through their chaperone activity [80].

ER stress, inflammation and oxidative stress pathways are linked in pathological conditions [81,82]. When ER stress persists, a terminal UPR program leads the cell to self-destruction. In fact, any intervention that alters basic cellular functions related to proteostasis, including protein synthesis, degradation or folding, has an impact on proteotoxicity and may reduce proteostasis collapse.

Chronic ER stress and defects in UPR signaling are emerging as key contributors to a growing list of human diseases, including CMD, NPD and neurodegenerative diseases [27,28]. The correct folding of proteins and in the formation of disulfide bonds, which determine the normal structure and function of many proteins, depend on the depletion by reductive stress of mitochondrial ROS. Levels of cellular disulfide bonds decrease in many cells when the mitochondrial oxidant production is inhibited [83]. Reductive stress leads to the loss of disulfide bond formation and induces the UPR of the ER. UPR pathways aim to restore homeostasis by activating genes involved in protein folding [84].

Epigenetic markers and their inheritance can contribute to proteostasis-related phenotypes determined by the response to stress of the ER across generations [26]. The epigenetic markers on ER stress might contribute to metabolic diseases. Hyperlipidemia, hyperhomocysteinemia, hyperglycemia and inflammatory cytokines trigger the response of the ER leading to the UPR activation (Figure 2). ER stress activates NF-κB of activated B cells and JNK, with downstream effects on inflammatory recruitment, phosphorylation of insulin receptor signaling intermediates (to worsen insulin resistance), lipogenesis and oxidative stress, thus contributing to metabolic syndrome, obesity and diabetes [85], which are risk factors for CMD.

The deficient expression of chaperones, protein quality control pathways and HSP is one of the pathophysiological pathways involved in heart diseases [29]. There are many protein aggregation cardiomyopathies, skeletal muscle myopathies and cataracts that have been related to reduction of stress and incorrect folding of proteins [32]. The ER-initiated apoptosis and UPR participate in the pathophysiology of various cardiovascular diseases, including ischemic heart disease, the development of atherosclerosis and plaque rupture. Apoptotic signaling, which includes induction of the pro-apoptotic transcriptional factor C/EBP homologous protein, activation of JNK and cleavage of caspase-12, is initiated by prolonged and severe ER stress [30,31]. Lipid metabolism is also modulated by activation of the UPR pathways by controlling the transcriptional regulation of lipogenesis. Excess adipose mass and obesity are a product of increased de novo lipogenesis and triglyceride storage in the adipose tissue, which are risk factors for cardiovascular disease [86].

ER stress may also contribute to neurodegeneration in a range of neurodegenerative disorders. Proteostasis collapse and the accumulation of proteotoxic aggregates comprise also a key signature of age-related human neurodegenerative diseases including Huntington’s disease, amyotrophic lateral sclerosis and Machado–Joseph disease [34,87]. The UPR involves an ER membrane-bound transcription factor known as the cAMP response element-binding protein (CREB3) protein, which changes the glucocorticoid response by modulating the expression of the glucocorticoid receptors, particularly in the brain. CREB3-deficient mice show a diminished stress response with low levels of anxiety and low circulating corticosterone levels. These mice also have decreased dendritic branching in the hippocampus, consistent with increased glucocorticoid receptor responses [88].

In summary, the ER is the subcellular organelle in which cellular proteins are folded. Many environmental factors stop the ability of cells to properly fold proteins and modify post-translationally secretory and transmembrane proteins, leading to ER stress and oxidative stress. The ER generates ROS during protein overload. When the stress situation that initiated the UPR pathways remains unresolved, it results in impaired redox homeostasis and oxidative stress via protein overload, influencing mitochondrial functions (Figure 2). ROS generated by the ER target ER-resident proteins, enzymes, chaperones and calcium channels (Figure 2). ER based-calcium channels release calcium into the cytosol. Increased cytosolic calcium and calcium entry into mitochondria stimulate mitochondrial metabolism to produce more ROS [83,84]. Among proteins that may be abnormally folded, the DNA repair machinery and the shelterin proteins that protect form telomere shortening may be included [89] (Figure 2), as will be discussed in the next section. The increased protein folding demand, calcium and ROS signaling integrate with UPR pathways and can potentially lead to inflammatory responses [83,84]. ER functioning may be epigenetically determined. Chronic ER stress is emerging as a key contributor to a growing list of human diseases, including CMD and NPD.

## 4. Nucleus

**Figure 3:**
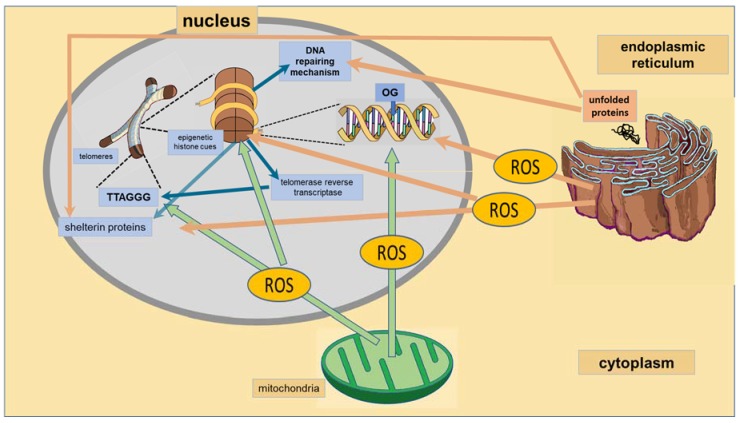
Epigenetic markers that lead to DNA transcription alterations and to telomere length determination. Blue arrows represent pathways to DNA and telomeres and to their regulatory mechanisms; green and brownish arrows represent the damaging effect of ROS produced by mitochondria and ER-altering DNA repairing mechanism, histone functioning and telomere length.

The nucleus is the organelle in which DNA is packed, forming highly organized complex coiled structures that finally form chromosomes. Chromosomes have at their ends, structures called telomeres. The length of telomeres is related to oxidative stress and may be epigenetically programmed. Oxidative stress is in part a consequence of mitochondrial activity and is increased when ER stress is present (Figure 3). ROS also cause malfunction in the nucleus including responses such as telomere shortening and damage to DNA repair proteins [89]. Chromosomal fusion, activation of DNA damage checkpoint responses, genome instability and impaired stem cell function are some of the consequences of telomere function loss, which may underlie the comorbidity of CMD and NPD. Pathways involved in DNA repair may be epigenetically programmed and may contribute to diseases. When ER stress is present, incorrect folding of proteins might include DNA repair enzymes, thus increasing DNA damage and telomere length, conserving enzymes that prevent telomere shortening.

### 4.1. Telomeres

Telomeres are regions of repetitive nucleotide sequences of TTAGGG associated with a complex of proteins known as shelterin that constitute the telomeric chromatin in human cells [36] (Figure 3). The ends of the chromosome are protected from deterioration or from fusion with neighboring chromosomes by these proteins. Chromosomal fusion, activation of DNA damage checkpoint responses, genome instability and impaired stem cell function are the consequences of the loss of telomere function. When telomeres shorten, the enzymes that duplicate DNA cannot continue their duplication up to the end of a chromosome, and therefore, the telomeric region is reduced in length by each replication. However, the telomeres are protected by shelterin protecting proteins, as well as by the RNA that encodes for telomeric DNA. Telomeres are replenished by the enzyme telomerase reverse transcriptase [36,37]. Telomere length shortening is age-related and is associated with oxidative stress [89].

Mammalian telomeres and sub-telomeric regions are organized in nucleosomes, which have many epigenetic modifications including DNA hypermethylation and trimethylation, which are a characteristic of heterochromatin. These chromatin modifications are important regulators of mammalian telomeres. Thus, telomeric chromatin is dynamic and re-programmable. These heterochromatin markers are used in the regulation of telomere length and structural integrity. The deletion of epigenetic regulators causes telomere-length control impairment and telomere shortening to an undesirable length [36,37].

In addition to methylation, telomeric regions and subtelomeric histone 3 and histone 4 are underacetylated. The heterochromatic state at telomeres and subtelomeres is determined by histone acetylation levels and causes telomere elongation and gene expression regulation at subtelomeres. Thus, histone acetylation is linked to telomere length stability [38].

Telomere length is influenced by personality traits and psychological symptoms. The mechanisms through which these psychological factors impact telomere length remain to be elucidated. However, individual differences in health behaviors, cardiovascular or inflammatory processes and acute stress responses do not appear to be the main pathways of action [90]. The long-term effect of chronic stress appears to accelerate telomere shortening and thus affect the psychological profile of individuals. Stress experiences lead to molecular alterations that result in reduced telomere length, accelerated cellular aging or epigenetic changes, thus affecting gene expression. Additionally, environmental influences modulate the effects of stress or pain on the genome by reducing resilience factors or increasing vulnerability factors of negative environmental influences. Epigenetic changes direct the expression of the genome across the lifespan and may shape vulnerability and resilience factors implicated in chronic pain conditions [40].

Early mortality and an increased risk of developing physical diseases typical of the elderly are associated with many neuropsychiatric illnesses. Several neuropsychiatric diseases such as major depressive disorder, bipolar disorder, post-traumatic stress disorder and possibly schizophrenia and anxiety disorders are associated with accelerated cellular aging, telomere length shortening in peripheral blood mononuclear cells and altered basal telomerase activity [91,92]. Stress during early stages of life and recent stress induce shortening of telomere length [93,94,95,96]. However, conflicting reports exist, and no conclusions can yet be made [39].

Among the emerging biological risk factors for CMD is shortened telomere length. Accelerated telomere shortening is associated with cardiovascular risk factors such as age, gender, obesity, smoking, sedentary life-style, excessive alcohol intake and even mental stress. Telomere shortening can often be caused by adopting an unhealthy lifestyle. Aging, degenerative disease and stress are potential regulators of telomere length. As with the aging process, CMD, most notably atherosclerosis, diabetes, as well as insulin resistance are found to be closely associated with telomere shortening, and patients with diabetes mellitus complications have a shorter leukocyte telomere length [97]. Almost all patients with coronary artery disease have shortened telomeres. Hypertrophic cardiomyopathy is associated with longer leukocyte telomere length, and studies investigating hypertension have reported both shorter and longer leukocyte telomere length than found in normotensive control subjects [41]. Studies have also shown that suppression of the oxidative stress slows down this shortening process. Telomere shortening was highly accelerated in the regions that were easily prone to atherosclerosis. Increased cellular turnover may result in cellular senescence associated with telomere shortening and may participate in coronary atherogenesis [97]. Furthermore, telomere length appears to be a potential biomarker of coronary artery disease [97] and cardiovascular aging [98]. However, pitfalls in the methodology of leukocyte telomere length quantification have limited its use as a marker [99].

### 4.2. DNA Reparation

DNA repair in the nucleus takes place via two main pathways: nucleotide excision repair (NER) and base excision repair (BER). Ultraviolet-induced and large DNA lesions induced by many chemicals are corrected by NER. This pathway has been better characterized than BER, and proteins participating directly or indirectly in this process have been described. Most of the oxidative lesions in DNA are repaired by BER, which constitutes a highly-conserved pathway throughout evolution. It is responsible for repairing most of the endogenous DNA damage including alkylations, oxidations, deaminations and depurinations, as well as single-strand breaks. Thus, BER removes frequently-produced lesions and maintains genomic integrity. Several enzymes participate in BER such as a glycosylase that initiates it, an apurinic/apyrimidinic-endonuclease or apurinic/apyrimidinic-lyase that cleaves the DNA backbone, polymerase b, the flap endonuclease and proliferating cell nuclear antigen [100].

Other pathways of DNA repair include: (a) *O*^6^-methylguanine DNA methyltransferase that repairs the naturally-occurring mutagenic DNA lesion consisting of *O*^6^-methylguanine substituting guanine, (b) mismatch repair and (c) double-strand break repair via homologous recombination and nonhomologous end joining. Positive and negative effects on lifespan and stress resistance have been found by elevating the level of expression of DNA repair genes in *Drosophila melanogaster*, depending on the genes overexpressed, the length and nature of stress, and, in some cases depending also on gender (sex) of the organism [101]. There is much less knowledge on the DNA repair pathways in mitochondria, although oxidative stress is present in them at a higher level [68,102].

The BER pathway is important for maintaining both the genetic stability and the methylation status of regions of DNA where a cytosine nucleotide is followed by a guanine nucleotide (CpG sites), which are central components of epigenetic regulation in vertebrates [42]. One of the most common DNA lesions produced by reactive oxygen species during oxidative stress is the oxidative modification of heterocyclic bases. One of these products is 8-oxo-7,8-dihydroguanine (OG). It produces an erroneous pairing with adenine that results in G to T and C to A substitutions in the DNA. It is corrected mainly by DNA 8-oxoguanine DNA glycosylase 1 (OGG1). OG was considered as an initiator of mutagenesis, but it is now considered as an epigenetic cue (Figure 3). When OG is present in G-rich regulatory elements in the promoters of some genes such as vascular endothelial growth factor, tumor necrosis factor α and *SIRT1*, it increases transcription via activation of the BER pathway [103]. Hence, BER substrates have recently been found to be epigenetic markers and to modulate transcription factor recognition/binding [43,103].

Repair mechanisms have been studied in neurons, especially to compare them to the mechanisms involved in rapidly reproducing cells such as cancer cells [104]. Damaged genomic DNA may contribute to the pathophysiology of different mental illnesses [105]. Decreased repair mechanisms in neurons are associated with aging and the coexistence of neurodegenerative diseases such as Alzheimer’s and Parkinson’s [104].

Different neuropsychiatric disorders show substantially high levels of oxidative DNA damage in the brain accompanied with morphological and functional alterations [105]. An elevated level of DNA damage was observed in patients with depression. Furthermore, single nucleotide polymorphisms of BER genes may modulate the risk of this disease [106]. DNA damage and DNA repair also participate in the etiopathology of schizophrenia and autism spectrum disorders [45]. Panic disorder is an anxiety disease characterized by sudden attacks of intense fear accompanied by high oxidative stress, which might induce DNA damage. However, no changes in DNA repair mechanisms have appeared in this disease; in particular, OGG1 remains unaltered in this disorder [107]. Exercise training influences brain function, the extent of neurogenesis and the expression of OGG1 and *SIRT1*. Exercise is also associated with increased hippocampal function. *SIRT1* level/activity is inversely correlate with OGG1 levels [108].

The discovery that BER is an active pathway for DNA demethylation in the brain provides a possibility by which previously pathological neurons may be reprogrammed to play a more favorable role [109]. BER proteins are involved in maintaining neuronal cell genome integrity, but there is inconclusive data regarding their role in providing protection against oxidative damage in neurodegenerative disorders [110]. Much hope is now being placed in agents targeting epigenetic processes [110].

Regarding CMD, DNA damage and oxidative stress coexist in the setting of coronary artery disease. Smoking, diabetes mellitus and other risk factors associated with atherogenesis could induce DNA damage [111,112]. Aggravation of the disease is caused by deterioration of DNA repair mechanisms. DNA strand breaks, mutations of single bases, modified bases (including oxidation) or DNA adducts are among the most common DNA damages found in this condition. There is also activation of DNA repair mechanisms including DNA strand break repair, BER and mismatch repair in the atherosclerotic plaques. DNA damage is present in all cells within the atherosclerotic plaque, including circulating cells and cells of the vessel wall. Plaque progression and instability might be promoted by double-stranded breaks and favor cell senescence, apoptosis and inflammation. DNA damage in vascular smooth muscle has little effect on atherogenesis, but it modifies plaque phenotype, and in advanced lesions, it inhibits fibrous cap areas [111,112]. Inhibiting DNA damage in atherosclerosis may be a novel target to promote plaque stability [96,113].

In addition, nuclear and mitochondrial DNA damage can be found in many inherited and acquired vascular diseases. Persistent DNA damage and lesions are found in multiple cell types. In turn, DNA repair mechanisms are activated by DNA damage in many vascular and endothelial diseases [46].

## 5. Possible Interventions to Promote the Modification of Epigenetic Cues that Alter Subcellular Functioning

In contrast to genetic mutations, the plasticity of epigenetic changes renders them potential targets for prevention or reversion by lifestyle, non-pharmacological and pharmacological interventions [9]. Several approaches that have been proposed for reversion of epigenetic cues in some CMD, particularly coronary artery disease and some NPD, in concrete stress-related disorders, have been recently described [9]. Epigenetics is a new challenge area for the development of new therapies that may return the chromatin to the state before being remodeling by environmental factors [12,13,14,15,16]. The reversing of histone modifications may help return altered phenotypes associated with diseases including NPD and CMD [114,115]. The use of natural compounds has been found to modify epigenetic cues [9]. Several drugs with a potential capacity to modulate the epigenetic machinery have been proposed [116,117,118,119,120,121,122,123]. These drugs modulate the activity of histone-modifying enzymes that are related to DNA methylation and could slow, stop or even revert the long-term effects that increase the risk for these diseases [124,125,126]. The area of study of epigenetic therapies is still in its beginnings, and novel effective alternatives will soon appear with the accumulation of experimental data and testing for clinical use.

## 6. Conclusions

Throughout human lifespan and particularly during early life, environmental factors determine epigenetic alterations that in turn define structural and functional changes in sub-cellular structures such as the mitochondria, the ER and the nucleus in cells composing different tissues. Epigenetic alterations in the nervous and cardiovascular systems might participate in the development of risk or resilience to NPD and CMD. Better knowledge of the beneficial or deleterious mechanisms induced by epigenetic alterations during early life upon sub-cellular structure functioning might allow for the development of better, more effective and safe preventive or therapeutic alternatives. Regarding this issue, several new pharmacological agents acting to revert or diminish the molecular alterations induced by environmental factors are currently being tested. Thus, epigenetic modulation of cellular organelles including mitochondrial biogenesis and function, of endoplasmic reticulum stress and of processes happening in the nucleus such as determination of telomere length and DNA repair might participate in the simultaneous increases in susceptibility or resilience to develop CMD and NPD.

## Figures and Tables

**Table 1:** Epigenetics in subcellular structures and possible related neuropsychiatric and cardiometabolic disorders.

Subcellular Structure		Epigenetics and the Structure	Related Disorders
Mitochondria		Crosstalk with nucleus: -Leaves epigenetic markers through the generation of metabolites by the tricarboxylic acid cycle [17,18,19]. -Regulates mitochondrial biogenesis through acetylation of FOXOs and PCG-1α [17,18,19].	-neurodegenerative diseases: Alzheimer’s disease [20].
			-metabolic diseases: type 2 diabetes mellitus [21,22].
			-cardiovascular diseases: myocardial ischemia, cardiomyopathy and heart failure [23,24].
			-psychiatric disorders: bipolar disorder, schizophrenia, autism, attention deficit-hyperactivity disorder [25].
Endoplasmic Reticulum		Expression of chaperones and heat shock proteins that prevent unfolding or misfolding of proteins is a target of epigenetic markers [26].	-metabolic diseases: metabolic syndrome, obesity and diabetes [27,28].
			-cardiovascular diseases: aggregation cardiomyopathies [29,30,31,32,33].
			-neurodegenerative diseases: Parkinson’s, Alzheimer’s and Huntington’s disease, amyotrophic lateral sclerosis and Machado–Joseph disease [34,35].
Nucleus	Telomeres	Telomeres are rich in epigenetic markers that determine their shortening [36,37,38].	-psychiatric disorders: chronic stress, pain, MDD, BD, PTSD, schizophrenia, anxiety disorders [39,40].
			-cardiovascular diseases: coronary heart disease, left ventricular hypertrophy [41].
	DNA Reparation	The BER pathway is important for maintaining both the genetic stability and the methylation status [42,43,44].	Psychiatric disorders; schizophrenia, autism spectrum disorders [45].
		The BER substrate, 8-oxoguanine, is an epigenetic marker modulating transcription factor recognition/binding [44].	Cardiovascular diseases: atherosclerosis, vascular smooth muscle cell dysfunction [46].

Abbreviations: FOXOs, forkhead transcription factors; PCG-1α, peroxisome proliferator-activated receptor gamma coactivator 1-alpha; MDD, major depressive disorder; BD, bipolar disorder; PTSD, post-traumatic stress disorder; BER, base excision repair.

## Abbreviations

Acetyl CoA	Acetyl Coenzyme A
AMPK	AMP-Activated Protein Kinase
ATP	Adenosine Triphosphate
BD	Bipolar Disorder
BER	Base Excision Repair
Ca^2+^	Calcium Ion
cAMP	Cyclic Adenosine Monophosphate
CMD	Cardiometabolic Disorders
CpG	Cytosine Phosphate Guanine
CREB	cAMP Response Element-Binding Protein
DNA	Deoxyribonucleic Acid
ER	Endoplasmic Reticulum
Fe-S	Iron-Sulfur Centers
FOXOs	Forkhead Transcription Factors
Hsp	Heat Shock Proteins
JNK	c-Jun N-Terminal Kinase
MDD	Major Depressive Disorder
mtPTP	Mitochondrial Permeability Transition Pore
NAD+	β-Nicotinamide Adenine Dinucleotide
NADH	Reduced Nicotinamide Adenine Dinucleotide
NER	Nucleotide Excision Repair
NF-κB	Nuclear Factor Kappa-Light Chain Enhancer of Activated B Cells
NPD	Neuropsychiatric Disorders
OGG1	8-Oxoguanine Glycosylase-1
PCG1α	Peroxisome Proliferator-Activated Receptor gamma Coactivator 1 Alpha
PKA	cAMP-Dependent Protein Kinase
PTSD	Post-Traumatic Stress Disorder
ROS	Reactive Oxygen Species
*SIRT1*	Sirtuin 1
UPR	Unfolded Protein Response
